# Budget impact analysis of venetoclax for the management of acute myeloid leukemia from the perspective of the social security and the private sector in Argentina

**DOI:** 10.1371/journal.pone.0295798

**Published:** 2024-01-04

**Authors:** Alfredo Palacios, Natalia Espinola, Juan Martin Gonzalez, Carlos Rojas-Roque, Maria Marta Rivas, Diego Kanevski, Pierre Morisset, Federico Augustovski, Andres Pichon-Riviere, Ariel Bardach

**Affiliations:** 1 Department of Health Technology Assessment and Health Economics, Institute for Clinical Effectiveness and Health Policy (IECS), Buenos Aires, Argentina; 2 Department of Economics, Universidad de Buenos Aires, Buenos Aires, Argentina; 3 Centre for Health Economics (CHE), University of York, York, United Kingdom; 4 Hospital Universitario Austral, Buenos Aires, Argentina; 5 AbbVie Argentina, Buenos Aires, Argentina; El Bosque University Faculty of Medicine: Universidad El Bosque Facultad de Medicina, COLOMBIA

## Abstract

**Objective:**

This study aimed to estimate the budget impact of the incorporation of venetoclax for the treatment of patients with Acute Myeloid Leukemia (AML) over 75 years of age or those with comorbidities and contraindications for the use of intensive chemotherapy, from the perspective of the social security and the private third-party payers in Argentina.

**Methods:**

A budget impact model was adapted to estimate the cost difference between the current scenario (azacitidine, decitabine and low doses of cytarabine) and the new scenario (incorporation of venetoclax) for a third-party payer over a time horizon of three years. Input parameters were obtained from a literature review, validated or complemented by expert opinion using a modified Panel Delphi approach. All direct medical costs were estimated by the micro-costing approach and were expressed in US dollars (USD) as of September 2020 (1 USD = 76.18 Argentine pesos).

**Results:**

For a third-party payer with a cohort of 1,000,000 individuals covered, incorporating venetoclax was associated with an average budget impact per-member per-month (PMPM) of $0.11 USD for the social security sector and $0.07 USD for the private sector. The duration of treatment with venetoclax was the most influential parameter in the budget impact results.

**Conclusion:**

The introduction of venetoclax was associated with a positive and slight budget impact. These findings are informative to support policy decisions aimed to expand the current treatment landscape of AML.

## Introduction

Acute Myeloid Leukemias (AMLs) represent a group of myeloid neoplasms with diverse etiology and genetic heterogeneity. These neoplasms result from a clonal proliferation of abnormal hematopoietic precursor cells with different degrees of differentiation, which infiltrate the bone marrow and sometimes other organs or systems, causing death by hemorrhage and/or infection. In the USA, annually 1.5 to 3 per 100,000 individuals are diagnosed with AML, representing 15 to 20% of acute leukemias in children and adolescents, and up to 80% in adults [1]. The median age at diagnosis is approximately 65 years and its incidence increases with age. The pathology is more frequent in men (male:female ratio of 5:3) and in non-Hispanic whites [2].

AML treatment consists of using different chemotherapeutic agents and their different combinations, during two phases: the induction phase, where remission is achieved, and the consolidation phase, where the risk of relapse is reduced. However, there are cases in which the standard induction regimen cannot be administered, given the comorbidities, functional status, or age of the patients [1]. These conditions imply greater morbidity and mortality during standard induction treatment; thus, its indication is not recommended. In these cases, it is recommended therapeutic alternatives, such as venetoclax (VEN) + hypomethylating agents, or VEN in combination with low dose cytarabine (LDC) [3]. Nowadays, there is no consistent evidence that more intensive regimens have better rates of complete remission [4].

VEN was approved by the Food and Drug Administration (FDA) in November 2018 for the treatment of AML, in combination with azacitidine, decitabine or LDC, for patients 75 years of age or older, or patients who due to their comorbidities cannot receive intensive chemotherapy [5]. In February 2016 VEN was approved for its use in AML by the European Medicines Agency (EMA) [6]. In Argentina, the National Administration of Drugs, Food and Medical Technology (ANMAT for its acronym in Spanish) approved VEN in April 2019 for AML treatment [7]. As the treatment landscape for AML is rapidly evolving with the approval of a number of novel therapies over the past 5 years [8], there are concerns about how the potential coverage of these treatments will financially impact the local healthcare systems.

Regarding coverage and reimbursement policies, the Canadian Agency for Drugs and Technologies in Health (CADTH) recommends that VEN in combination with azacitidine should be reimbursed for the treatment of patients with newly diagnosed AML who are 75 years or older, or who have comorbidities that preclude use of intensive induction chemotherapy, only if certain conditions are met [9]. In the United Kingdom National Health System (NHS), the National Institute for Health and Care Excellence (NICE) recommended the coverage of VEN only if the company provides a commercial arrangement [10]. In the US, the available evidence suggest that venetoclax plus azacitidine was deemed a cost-effective strategy for treating AML patients ineligible for intensive chemotherapy from a third-party payer perspective [11, 12], and combining venetoclax with azacitidine or low-dose cytarabine resulted in significantly lower costs per patient achieving remission compared to using azacitidine or low-dose cytarabine alone [13].

In Argentina, the National Commission on Health Technologies of Health (CONETEC) conducted a HTA report with a budget impact analysis that suggest that the incorporation of venetoclax in the treatment of AML would imply an relative increase in the budget per patient of 26%, representing a absolute budget impact per patient of AR$ 3,830,841 (USD 47,126) per year [14]. However, this technical report did not follow the recommendations of the ISPOR Task Force on good research practices for budget impact analysis in health [15], i.e. the perspective of the analysis is not totally clear, the reports does not present the unit costs used in the analysis, do not include scenario or sensitivity analysis, among other limitations that could affect the accuracy of the estimations and the replicability of the analysis for different local third-party payers.

Currently, the Argentine health system is characterized by decentralization and fragmentation in its social insurance mechanisms. Health service coverage is fragmented into three subsectors: the public subsector (national, provincial, and municipal), the social security subsector (*Obras Sociales*), and the private insurance subsector [16, 17]. According to the 2010 Population Census, 37.9% of the population are covered exclusively by the public subsector; 46.4% had coverage of social security; and private insurance was accessed by 15.7% of the population [18]. Social security, the most important subsector, is organized around three large groups: i) 269 *Obras Sociales Nacionales* (OSNs); ii) 24 *Obras Sociales Provinciales* (OSPs); and iii) the National Institute of Social Services for Retirees and Pensioners (*INSSJyP–PAMI*, acronym in Spanish). The private insurance sector involves approximately 200 private insurance or prepaid health plans [16, 17].

This study aimed to estimate the budget impact of VEN potential coverage for the treatment of patients with AML over 75 years of age, or those with comorbidities that contraindicate the use of intensive chemotherapy, from the perspective of the social security and the private insurance third-party payers in Argentina.

## Methods

### Model structure

The budget impact model was developed in Microsoft Office Excel (Microsoft Corporation, Redmond, WA, USA) and was evaluated, adapted and validated for its use in the Argentinean context. The budget impact model considers a hypothetical third party-payer with 1,000,000 covered individuals to estimate two scenarios: the current scenario (without VEN) and the new, projected scenario (incorporation of VEN). A comparison between the current and new scenarios provided an estimate of the budget impact of VEN over a 3-year time horizon. The budget impact results were presented in absolute and relative terms and per-member per-month (PMPM). This study followed the ISPOR Task Force reporting Budget Impact Analysis in health [15].

**Fig 1:**
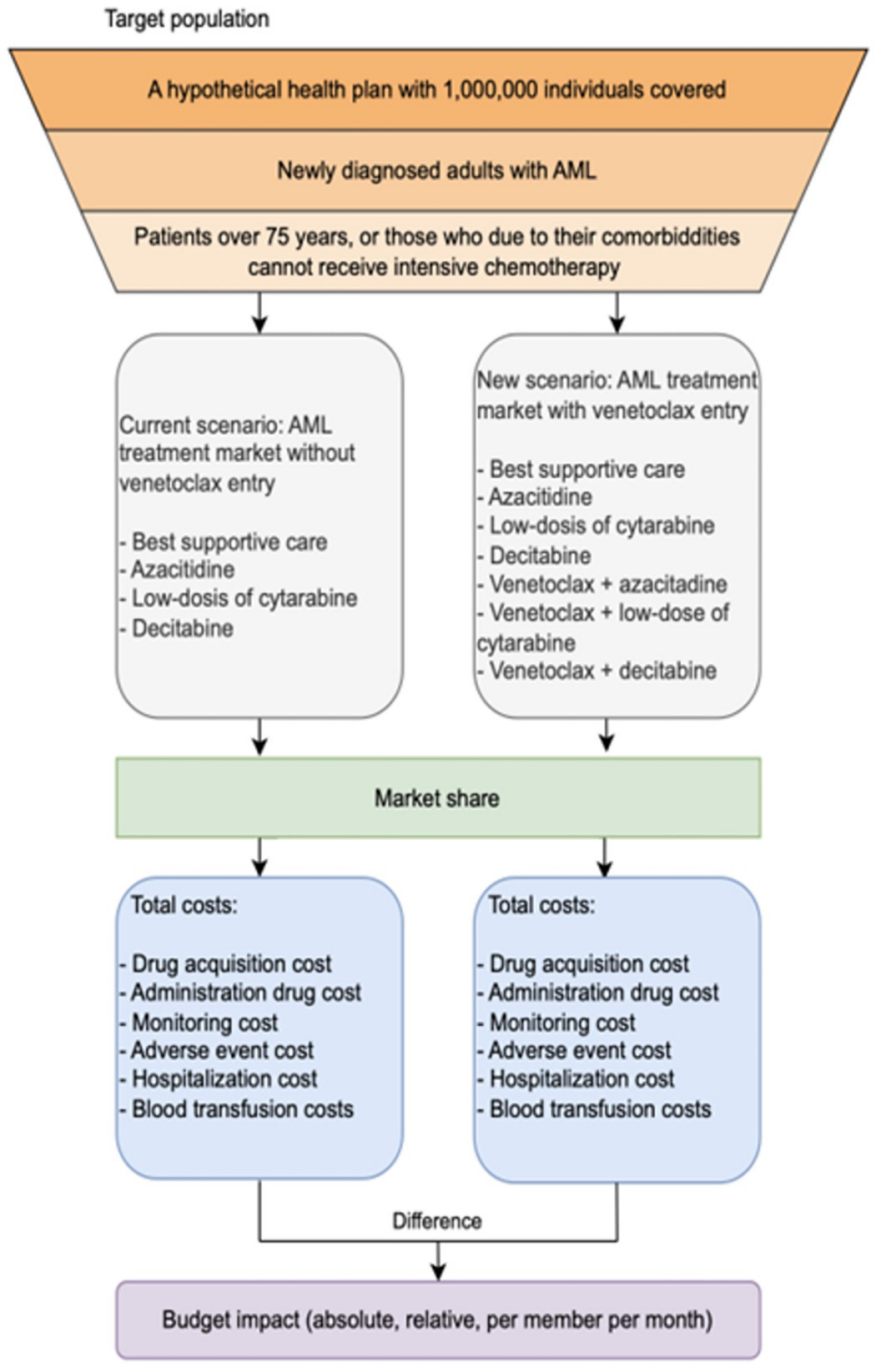


The analytical structure of the model consists of three main components that combined give the estimation of the budget impact of VEN for a third-party payer: i) the estimation of the target population to receive the drug; ii) the market share of chemotherapy drugs (current and new scenarios with the incorporation of VEN), iii) the acquisition costs of the chemotherapy drugs, and the direct medical costs associated to the health condition (disease management, hospitalizations, blood transfusions, etc.). Fig 1 presents the analytical framework of our budget impact model.

### Intervention and comparators

For the intervention, we defined the following combinations of VEN: venetoclax + azacitidine (VEN + AZA), venetoclax + LDC (VEN + LDC), venetoclax + decitabine (VEN + DB). The comparators were the following: best supportive care (BSC), azacitidine (AZA), LDC and decitabine (DB). We included the comparators based on drugs commercially available in Argentina, the modified Delphi panel suggestions and previous studies [12, 19].

### Model assumptions

The model assumes that patients transition through two stages during the first budget year: the active treatment stage and the active post-treatment stage. The active treatment stage comprises the period from the start of the administration of the therapies under study (VEN and comparators) to their completion. The duration of the active treatment stage depends on the observed clinical response. The active post-treatment stage comprises the period after the active treatment stage and before the end of the first budget year. During this stage, it is assumed that patients do not receive the administration of any therapies under analysis, but instead receive the best supportive care. The model was developed in 28-day cycles (4-week cycles). All cost categories, except those linked to adverse events (which are assumed to occur only once during the treatment period), were considered separately for each of these periods.

We assume that the prevalence of AML is constant through the 3-year time horizon of the model. For the current scenario, we assume a constant market share for each regimen of treatment. Lastly, due to the short time horizon, we do not consider inflation in the outcomes of the model.

### Target population

As mentioned in the introduction section, VEN has been approved for the treatment of AML in combination with azacitidine, decitabine or LDC, for patients 75 years of age or older, or patients who due to their comorbidities cannot receive intensive chemotherapy. Due to the limited availability of local studies to retrieve the epidemiological parameters necessary for the estimation of the target population for VEN, we conducted an international literature review to identify values for these parameters. However, some assumptions were necessary to characterize the candidate population to receive VEN. The most important of them was to assume that the AML individuals aged 65 or older could be a proxy of the "patients 75 years of age or older, or patients who due to their comorbidities cannot receive intensive chemotherapy". This assumption was discussed and validated locally by the modified Delphi panel.

Table 1 reports the epidemiological parameters. The incidence rate of AML per 100,000 individuals aged 65 years or older (20.1 cases) was retrieved from the National Institute of Cancer in the US [20]. The percentage of patients with no indication for standard induction treatment (64%) was retrieved from Dombret et al. [21] and Mela-Osorio et al. [22].

**Table 1:** Summary of epidemiology, efficacy and security parameters for the budget impact model.

Parameters	Regimen					
	AZ	DB	LDC	VEN+AZ	VEN+DB	VEN+LDC
*Epidemiology*						
Incidence rate of AML	20.1 cases per 100,000 individuals aged 65 years or older. Source: based on the National Cancer Institute (2014)					
Percentage of patients with no indication for standard induction treatment	64%. Source: retrieved from Drombret et al. (2015) and Mela-Osorio et al. (2019)					
*Efficacy*						
Complete remission (CR) or CR with incomplete recovery of blood count, in days	27.8	25.62	10.7	66.4	74.2	48
Mean time for achieving recovery of blood count, in cycles of 28 days each	2.89	6.74	5.8	2.04	2.98	2.2
% of patients achieving transfusion independence in platelet or red blood cells for 56 days	38.5	30	16.7	59.8	52	41
Mean duration of the active treatment, in cycles of 28 days	8.8	6.9	3.76	10.98	10.94	7.06
Source	Dombret et al. (2015)	Kantarjian et al. (2012)	Kantarjian et al. (2012)	Di Nardo et al. (2020)	Pollyea et al. (2018)	Wei et al. (2019) and Wei et al. (2020)
*Security*						
Neutropenia	26.3%	32.0%	20.0%	42.0%	38.0%	46.0%
Febrile neutropenia	28.0%	32.0%	25.0%	42.0%	65.0%	32.0%
Thrombocytopenia	23.7%	40.0%	35.0%	45.0%	45.0%	45.0%
Anemia	15.7%	34.0%	27.0%	26.0%	15.0%	25.0%
Hypokalemia	5.1%	11.0%	9.0%	11.0%	16.0%	28.0%
Pneumonia	19.1%	21.0%	19.1%	20.0%	32.0%	20.0%
Source	Dombret et al. (2015)	Kantarjian et al. (2012)	Kantarjian et al. (2012)	Di Nardo et al. (2020); Wei et al. (2019); Wei et al. (2020); Pollyea et al. (2018)		

Notes: BSC: best supportive care; AZA: azacitidine; DB: decitabine; LDC: low-dose cytarabine; VEN: venetoclax.

### Efficacy and safety parameters

Efficacy parameters include the complete remission, the mean time for achieving recovery of blood count, the percentage of patients achieving transfusion independence and the mean duration of the active treatment. The efficacy and safety parameters were retrieved from published literature [4, 21, 23–28].

### Market share

Market shares before and after the entry of VEN were obtained from projections provided by AbbVie Inc., Argentina, and validated by the modified Delphi panel. We assumed that the market shares for each technology are equal for both perspectives (social security and private third-party payers). The market shares for each technology are available in the S1 Table.

### Direct medical costs

The direct medical costs considered in the model were classified into the following categories: i) drug acquisition costs; ii) healthcare costs, and iii) adverse events costs. These costs were originally reported in Argentinian pesos (ARS) and then converted to US dollars (USD) as for September 2020 (1 USD = 76.18 ARS) [29].

i) Drug acquisition costs were obtained from public databases that report the retail price of drugs marketed in Argentina [30]. We used the most recent retail price at the moment to perform the analysis (September 2020) and we converted the retail prices to ex-factory prices by applying the conversion factor suggested by the Argentinian Ministry of Economy [31]. For each drug, the total acquisition cost was estimated from the ex-factory price, the dosage of the therapy administered and the mean duration of treatment. For all drugs, we assumed there was no wastage. Table 2 presented the dosage, pack size, ex-factory price per pack, and the patient-year total drug cost. A detailed description of these estimations is presented in the S2 Table.

**Table 2:** Acquisition drug costs by regimen. Costs expressed in US dollars (USD, $), 2020.

Regimen	Dosage	Pack size	Ex-factory price per pack	Patient-year total drug cost
AZA	75 mg/m^2^/per-day, days 1–7	100 mg * 1	$1,069	$134,662
LDC	20 mg/m^2^/per-day, days 1–5	50 mg * 1	$6	$6,682
DB	20 mg/m^2^/per-day, days 1–10	100 mg * 1	$1,250	$47,430
VEN + AZA	Initial treatment: VEN + AZA 100mg, 200mg, 400mg for day 1, 2 and 3. Post-initial treatment and post-active treatment: VEN + AZA 400 mg/per-day. AZA 75 mg/m^2^/per-day, days 1–7	VEN: 100 mg * 120 AZA: 100 mg *1	VEN = $5,522 AZA = $1,069	$186,207
VEN + DB	Initial treatment: VEN + DB 100mg, 200mg, 400mg for day 1, 2 and 3. Post-initial treatment and post-active treatment: VEN + DB 400 mg/per-day. DB 20 mg/m^2^/per-day, days 1–10	VEN: 100 mg * 120 DB: 500 mg *1	VEN = $5,522 DB = $1,250	$90,262
VEN + LDC	Initial treatment: VEN + LDC 100mg, 200mg, 400mg, 600mg, day 1, 2, 3 and 4. Post-initial treatment and post-active treatment: VEN + LDC 600 mg/per-day. LDC 20 mg/m^2^/per-day, days 1–5	VEN: 100 mg * 120 LDC: 100 mg *1	VEN = $5,522 LDC = $6	$20,999

Notes: BSC: best supportive care; AZA: azacitidine; DB: decitabine; LDC: low-dose cytarabine; VEN: venetoclax.

ii) The healthcare costs were estimated using the micro-costing approach. Local experts carried out the identification and measurement of health resources used for each treatment through the modified Delphi panel. Only intravenous treatments (IV) and subcutaneous treatments (SC) were assumed to incur administration costs. For IV drugs, we assumed that during the first two cycles the patients receive the treatment under hospitalization. For subsequent cycles, the cost of administration was estimated based on a daily stay at the hospital. For the SC administration, we assumed the cost was equal to a home nursing visit. For the hospitalization costs, we estimated the cost of the neutropenic hospital room. The total cost of transfusions associated with each treatment scheme was obtained by a weighted average of the percentage of patients who achieve transfusion independence or not, the unit cost of transfusions, the duration of transfusion independence and the number of transfusions. The rate of use of transfusions was obtained through the opinion of a local expert and later was validated by the modified Delphi panel. Further information about the healthcare resources used during the monitoring as well as the rate of use of transfusions can be found in the S3 and S4 Tables.

iii) For each AE we estimated weighted costs by multiplying the probability of each AE by the unit cost for each AE. The probability of each AE was obtained from published literature [4, 21, 23–26] while the identification and measurement of healthcare resources were carried out by literature review [4, 21, 23–26] and validated by a local expert. The costs for the healthcare resources for each cost category as well as for the adverse events are reported in Table 3.

**Table 3:** Healthcare resource costs according to the cost category. Costs expressed in US dollars (USD, $), 2020.

Cost category/Healthcare resource	Perspective	
	Social security	Private sector
*Administration*		
Intravenous administration	$183	$206
Subcutaneous administration	$3	$3
*Monitoring*		
Hemogram	$2	$2
Chemical panel*	$29	$34
Bone marrow aspiration	$109	$114
Bone marrow biopsy	$150	$164
*Hospitalization*		
Hospitalization in neutropenic room	$226	$381
*Blood transfusions*		
Red blood cells	$121	$140
Platelets by apheresis	$278	$379
*Adverse events*		
Neutropenia**	$0	$0
Febrile neutropenia	$2,529	$2,773
Thrombocytopenia	$290	$396
Anemia	$105	$133
Hypokalemia	$60	$60
Pneumonia	$1,843	$2,035

Notes:

*Chemical panel include the following tests: renal function, creatinine clearance test, bilirubin test, liver function tests, electrolytes, calcemia, phosphatemia, serum uric acid, total protein, glycemia, serum albumin, LDH, serum chloride, bicarbonate.

**The cost for the management of neutropenia is already included in the hospitalization in the neutropenic room.

The source for the unit costs for each healthcare resource and AE and perspective (social security and private third-party payer) came from the Institute of Clinical Effectiveness and Health Policy (IECS) Unit Cost Database [32].

### The modified Delphi panel

We conducted a modified Delphi panel to validate or adapt the model’s structure and the parameters required to populate the budget impact model. The modified Delphi panel comprised six expert onco-hematologists with vast clinical experience in assisting adult patients with AML in the social security and private sectors in Argentina. The participants of the modified Delphi panel were selected based on their clinical experience. The participants gave written informed consent for research participation. The modified Delphi panel has two rounds. Both rounds took place in person at IECS in November 2019. The electronic version of the questionnaire used in the modified Delphi panel is available in Spanish and can be consulted in the S1 and S2 Files.

### Sensitivity analysis

To evaluate the effect of uncertainty associated with the parameters of the model on the budget impact results, deterministic (one-way) sensitivity analyses (DSAs) were performed. The parameters varied from their default values by their 95% confidence interval or, when the confidence interval was not available, by +/-25%, of their base case values [33, 34].

### Scenario analysis

We performed different scenario analysis to assess the impact of different population pyramids in the per-member per-month budget impact. The percentage of individuals aged 65 years or over varied from 5% (a population pyramid similar to the private sector of Argentina), to 10% (a population pyramid similar to the entire health system of the country), 15% and 20% (population pyramid similar to the social security sector) [18] and up to 78% (a similar population pyramid reported in the national social health insurance fund for retired workers (Programa de Asistencia Médica Integral [PAMI]) [35].

### Model validation

The model structure and calculations were reviewed and validated by academic experts from IECS. All input parameters were initially reviewed and validated by a local onco-hematologist expert and then by five experts through the modified Delphi panel. Suggestions for revision and/or adaptation were addressed before conducting the analysis.

## Results

### Target population

For a third-party payer with a 1,000,000 covered population, the target population was composed of 129 individuals by year. This corresponds to the multiplication of the covered population by the incidence rate by the percentage of chemotherapy (= 1,000,000*0.000201*0.64). After the introduction of VEN, 57, 76 and 80 patients will receive VEN in its different combinations during year 1, year 2 and year 3, respectively. VEN+AZA will be the most prevalent combination, with 26, 45 and 49 patients in year 1, year 2 and year 3. Further details can be found in the S5 Table.

### Budget impact results

Table 4 shows the total budget impact of VEN coverage for a third-party payer with a 1,000,000 covered population and a target population of 129 patients per year.

**Table 4:** Absolute budget impact for the current (without VEN) and projected scenario (with VEN) for a third-party payer with a 1,000,000 covered population and a target population of 129 patients per year. Costs expressed in US dollars (USD, $), 2020.

	Social Security			Private Sector		
	Year 1	Year 2	Year 3	Year 1	Year 2	Year 3
*Cost component*	Current scenario without VEN (A)					
Drug	$11,143,997	$11,143,997	$11,143,997	$11,143,997	$11,143,997	$11,143,997
Administration	$193,294	$193,294	$193,294	$231,543	$231,543	$231,543
Adverse events	$122,748	$122,748	$122,748	$137,453	$137,453	$137,453
Hospitalization	$5,287,818	$5,287,818	$5,287,818	$8,916,164	$8,916,164	$8,916,164
Monitoring	$163,806	$163,806	$163,806	$187,833	$187,833	$187,833
Blood transfusion	$2,685,672	$2,685,672	$2,685,672	$3,538,762	$3,538,762	$3,538,762
Total	$19,597,335	$19,597,335	$19,597,335	$24,155,752	$24,155,752	$24,155,752
*Cost component*	Projected scenario with VEN (B)					
Drug	$11,046,855	$12,920,459	$13,432,047	$11,046,855	$12,920,459	$13,432,047
Administration	$253,177	$266,046	$270,833	$303,362	$318,735	$324,461
Adverse events	$158,589	$168,570	$170,594	$177,804	$189,049	$191,324
Hospitalization	$4,853,804	$4,601,535	$4,533,200	$8,184,341	$7,758,974	$7,643,749
Monitoring	$168,199	$169,250	$169,347	$192,388	$193,288	$193,333
Blood transfusion	$2,664,634	$2,653,930	$2,651,133	$3,510,187	$3,495,625	$3,491,816
Total	$19,145,257	$20,779,790	$21,227,154	$23,414,938	$24,876,129	$25,276,730
*Cost component*	Budget impact (B-A)					
Drug	-$97,142	$1,776,462	$2,288,050	-$97,142	$1,776,462	$2,288,050
Administration	$59,883	$72,753	$77,539	$71,819	$87,192	$92,918
Adverse events	$35,841	$45,822	$47,846	$40,351	$51,596	$53,871
Hospitalization	-$434,014	-$686,283	-$754,618	-$731,822	-$1,157,190	-$1,272,415
Monitoring	$4,393	$5,444	$5,540	$4,555	$5,455	$5,500
Blood transfusion	-$21,039	-$31,743	-$34,539	-$28,575	-$43,137	-$46,946
Total	-$452,078	$1,182,455	$1,629,818	-$740,814	$720,377	$1,120,977

For the social security third-party payer, VEN incorporation was associated with financial savings of $452,078 USD in year 1, and a budget impact of $1,182,455 USD and $1,629,818 USD for year 2 and 3, respectively. For the private sector third-party payer, VEN coverage was associated with financial savings of $740,814 USD in year 1, and the budget impact increased to $720,377 USD and $1,120,977 USD for year 2 and year 3.

On average, the budget impact share in the total budget was estimated at 4.01% for the social security third-party payer and 1.52% for a private sector third-party payer. Financial savings were reported in the detailed budget impact analysis, driven mainly by hospitalizations and blood transfusion costs. Drug acquisition costs and hospitalization costs represented nearly 60% and 22% of the total impact budget, respectively. These results are valid for both sectors.

**Fig 2:**
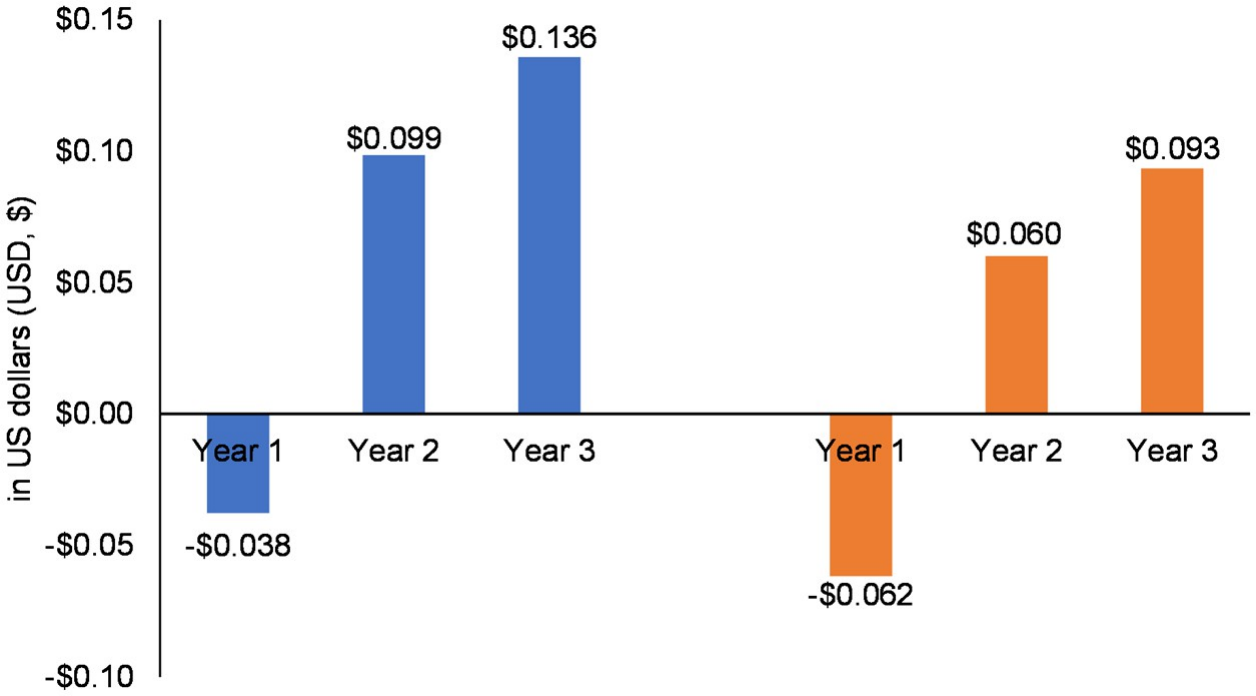


In Fig 2 we reported the current and the projected per-member per-month (PMPM) budget impact associated with the addition of VEN for the first-line treatment in previously untreated patients. The introduction of VEN resulted in cost savings of $0.038 USD PMPM and $0.062 USD PMPM in the first year for the social security and private sector, respectively.

### Sensitivity analysis

**Fig 3:**
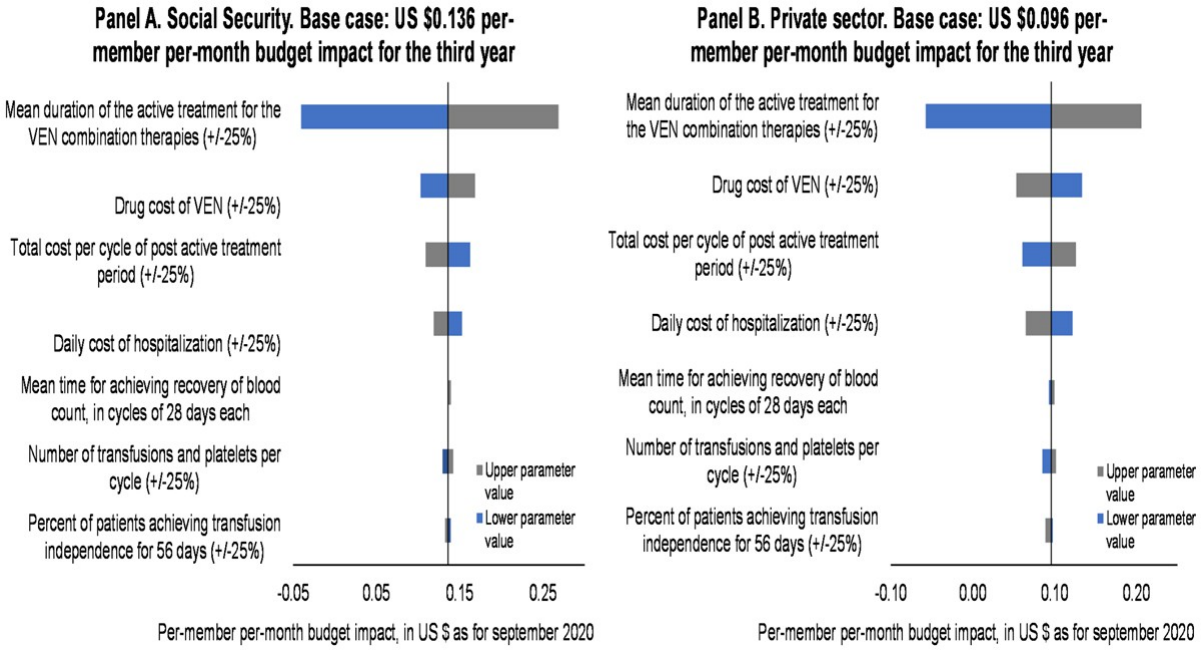


The one-way sensitivity analysis is depicted in Fig 3. For both perspectives, the mean duration of the active treatment for the VEN combination therapies is the parameter that has the most impact on the per-member per-month budget impact for the third year. When the duration of the active treatment was reduced by 25%, VEN was associated with a saving in the per-patient per-year budget impact for the third year, in both perspectives. On the other hand, when we increased 25% in the duration of the active treatment for the VEN combination, the per-member per-month budget impact for the third year was more than $0.27 USD for the social security perspective and $0.21 USD for the private sector. The rest of the parameters have a lower impact on the per-member per-month budget impact for the third year.

Table 5 shows the results of different third-party payer pyramid populations on the budget impact per-member per-month. For the social security perspective, when the percentage of individuals 65 years or older is 5%, the associated budget impact per-member per-month is $0.007. When the percentage of individuals aged 65 years or older is 78%%, the budget impact per-member per-month is $0.105. For the private sector, the budget impact per-member per-month is quite similar to the reported for social security.

**Table 5:** Budget impact per-member per-month for the year 3, according to different population pyramids, for the social security and the private insurance sector. Costs expressed in US dollars (USD, $), 2020.

Perspective	Percentage of individuals 65 years or over				
	5%	10%	15%	20%	78%
Social Security	$0.007	$0.014	$0.020	$0.027	$0.105
Private sector	$0.005	$0.009	$0.014	$0.019	$0.073

## Discussion

This study estimated the budget impact of the coverage of VEN for the treatment of patients with AML from the perspective of the social security and the private third-party payers in Argentina. Our findings show that for the social security third-party payer, VEN incorporation was associated with financial savings of $0.038 USD PMPM in year 1 to a budget impact PMPM of $0.136 USD for year 3. For the private sector third-party payer, VEN coverage was associated with financial savings of $0.059 USD PMPM in year 1 to a budget impact of $0.096 USD PMPM for year 3. For both perspectives, the introduction of VEN was associated with an increment in the following cost categories: drug acquisition, drug administration and adverse events. However, our findings showed a net saving especially for the hospitalization costs and to a lesser extent by the transfusions, driven by the better efficacy of VEN combination therapies.

The results of the deterministic sensitivity analysis suggest that the parameter that has the greatest influence on the budget impact PMPM was the duration of treatment with venetoclax. A variation of +/-25% in the mean duration of VEN treatments translated into a net budget impact PMPM that can range approximately between $-0.04 USD and $0.27 USD for the social security perspective, while the values range between $-0.06 USD and $0.21 USD for the private sector. This result is not only explained by the higher acquisition cost of VEN associated with a longer duration of treatment, but also by the consequent higher acquisition cost of complementary therapies, in particular azacitidine. The duration of treatment also impacts the antifungal therapy since the recommended dose for those patients under VEN treatment is lower compared with patients under a different treatment, which ultimately impact the costs and budget.

In addition, we found an increasing financial burden for the third-party payer associated with serving populations with a higher proportion of elderly persons. When we assessed a pyramid population similar to the nationwide social health insurance fund for retired workers (PAMI, with 78% of the population above 65 years old), we found that the budget impact PMPM increases up to $0.11 USD for the social security and $0.07 USD for the private sector. On the other hand, in the case that 5% of the population were over 65 years of age (pyramid population similar to the average of the private health sector), the budget impact PMPM decreases to $0.01 USD and $0.005 USD for the social security and private sector, respectively.

As we mentioned in the introduction section, our study presents several strengths in comparison to the Argentinean’s CONETEC budget impact analysis, with some additional advantages valid to mention. First, our study considers a more comprehensive AML target population. While CONETEC’s study only considers AML patients older than 75 years old, our analysis also includes those patients with comorbidities that restrict the use of intensive chemotherapy. Second, our BIA considers diverse third-party payers perspectives that are relevant for the national healthcare system, in particular, the social security subsector (which includes PAMI), and the private insurance subsector, and our scenario analysis considers different pyramid population possibilities for each third-party payer Third, as Argentina has a fragmented health system, there isn’t a "unique" drug acquisition cost. Each third-party payer obtains its own acquisition cost. For this reason, the applied health economics studies conducted in the country could follow two approaches [36, 37]: i) to use the retail price (PVP) or ii) to use the ex-factory price that is calculated as the PVP divided by 1.7545. The latest could be considered a more "realistic" approach, closer to the actual prices paid by different third-party payers. However, CONETEC used the first approach, while our study follows the second and more realistic option. Fourth, the analysis performed by CONETEC did not include any other cost beyond the acquisition cost of the drug, so some savings items such as transfusions and hospitalizations were not factored in the budget impact analysis. Finally, one topic that was not considered in the CONETEC’s study was the dose reduction for venetoclax due to the antifungal therapy. The item was included in the product label and supported by the specialists who were part of the Delphi Panel. This has a consequence in the reduction of venetoclax costs.

Our study has some additional strengths. First, we adapted a budget impact model considering the characteristics of the local health system and clinical practice in relation to the management of the disease and the use of the technologies under study. Second, the research team developed an exhaustive search strategy for estimating epidemiological parameters, use of resources, and costs at the local level for both perspectives (social security and the private insurance subsector), with the advice of a local expert onco-hematologist. Likewise, all the estimates of costs of health events were made through the micro-costing method, which involved identifying health resources, their quantities, rates of use and unit costs. This made it possible for the study to have rigorous and well-founded estimates of cost parameters at the local level. Forth, those parameters in which solid evidence was not identified at the local level (epidemiological parameters, market shares of technologies and consumption of health resources) were validated or modified through the consensus of a group of six expert local onco-hematologists using the modified Delphi Panel methodology. Finally, the budget impact model included a robust deterministic sensitivity analysis and an alternative scenario analysis that allowed accounting for the uncertainty associated with the findings.

The study has some limitations. First, the evidence on the mean duration of treatment with VEN reported in the pivotal studies could differ from the reality of the different healthcare contexts in Argentina. As this parameter showed strong implications for the final budget impact result, our results must be interpreted in light of the reality of each third-party payer at the local level. Second, the simplifying conceptualization of the transition of patients between the stages of active treatment and active post-treatment may not reflect the usual care standards for the disease, given that in medical practice it could continue administering the therapy with which an adequate response was achieved during the active treatment period, beyond the budget period under analysis. Third, there is still no evidence of the cost-effectiveness of VEN at the local level, so the decision-making regarding the introduction of VEN would be limited to the budget impact results provided by this study. Therefore, it is important to explain that it would be of great importance to have a cost-effectiveness study of the technology at the local level that contemplates the health benefits (utilities, life years gained, quality-adjusted life years gained) in the long term. Finally, the current macroeconomic conditions in Argentina lead us to consider the results presented here with caution and to pay special attention to the evolution of the relative prices of VEN and its comparators and to the clinical management of the disease (mainly in relation to hospitalizations).

Drawing on Argentina’s challenges related to high-inflation rates, we followed the approach used in other local BIAs [36, 38] and decided to exclude inflationary factors from our results. It is important to mention that the ISPOR Task Force guidelines for BIA recommended inflation adjustments but did not provide specific guidance about how to handle this issue in practice. In our view, high inflation rates can distort BIA results, masking the genuine impact of technology coverage and intertwining it with the effects of inflation. In addition, since information on inflation in the future should be based on predictions, this involves an additional source of uncertainty on the results. By presenting our results without adjusting for inflation, we aim to provide a clearer and more straightforward view of the direct consequences of the technology under evaluation.

## Conclusion

The incorporation of VEN, a therapy now recommended by evidence-based guidelines for the elderly population with AML and nowadays is the standard of care in Argentina, was associated with a slight cost increase for the social security and private sector. These findings are informative to support policy decisions aimed at expanding the current treatment landscape of AML.

## Supporting information

S1 Table Market share for treatment regimen. Values are presented as a percentage.
(DOCX)

S2 Table Total drug cost estimation in active treatment period and post-active treatment period. Values expressed in $ dollars 2020.
(DOCX)

S3 Table Rate of use per-cycle and per-year for the healthcare resources used for the monitoring, according to the regimen. (DOCX)

S4 Table Rate of use per cycle for the transfusions. (DOCX)

S5 Table Distribution of the target population for the current and projected scenario. (DOCX)

S1 File Questionnaire (in Spanish) for the modified Delphi Panel. (DOCX)

S2 File General document of the declaration of conflicts of interest signed by participants in modified Delphi Panel. (DOCX)
