# Supplementary material for: Budget impact analysis of venetoclax for the management of acute myeloid leukemia from the perspective of the social security and the private sector in Argentina
Source: PLoS One. 2024 Jan 4;19(1):e0295798. doi: 10.1371/journal.pone.0295798 (PMC10766175; doi:10.1371/journal.pone.0295798)
Supplement: S2 Table — Values expressed in $ dollars 2020. (DOCX) [file pone.0295798.s002.docx]

*PLOS ONE*

**Budget impact analysis of venetoclax for the management of acute myeloid leukemia from the perspective of the social security and the private sector in Argentina**

Alfredo Palacios,^1,2,3^ Natalia Espinola,^1^ Juan Martin Gonzalez,^1^ Carlos Rojas-Roque,^1,3^ Maria Marta Rivas,^4^ Diego Kanevski,^5^ Pierre Morisset,^5^ Federico Augustovski,^1^ Andres Pichon-Riviere,^1^ Ariel Bardach^1^

^1^ Department of Health Technology Assessment and Health Economics, Institute for Clinical Effectiveness and Health Policy (IECS), Buenos Aires, Argentina

^2^ Department of Economics, Universidad de Buenos Aires, Buenos Aires, Argentina

^3^ Centre for Health Economics (CHE), University of York, York, UK

^4^ Hospital Universitario Austral, Buenos Aires, Argentina

^5^ AbbVie Argentina, Ing. Enrique Butty 240, C1106 CABA, Argentina

**Corresponding author**

Alfredo Palacios, MSc

Department of Health Technology Assessment and Health Economics, Institute for Clinical Effectiveness and Health Policy (IECS), Buenos Aires, Argentina

Department of Economics, Universidad de Buenos Aires, Buenos Aires, Argentina

Centre for Health Economics (CHE), University of York, York, UK

Email: alfredo.palacios@york.ac.uk

ORCID: 0000-0001-7684-0880

**Declarations**

**Conflicts of interest/Competing interests**

I have read the journal's policy and the authors of this manuscript have the following competing interests. Alfredo Palacios, Natalia Espinola, Juan Martin González, Carlos Rojas-Roque, Andrés Pichon-Riviere, Federico Augustovski and Ariel Bardach declare that they have no conflicts of interest. Diego Kanevsky and Pierre Morisset are employees of Abbvie and may own Abbvie stocks. Maria Marta Rivas has received speaker fees from Abbvie. This does not alter our adherence to PLOS ONE policies on sharing data and materials.

**Data Accessibility Statement**

All parameters used to populate the budget impact model are provided within the main manuscript and its supplementary material. The budget impact model itself will be made available upon reasonable request.

**Consent for publication**.

Not applicable.

**Acknowledgements.**

The authors wish to thank Isolda Fernandez, Mariela Gómez, Hernán Dick, Laura Fischman and Irene Rey, who participated in the modified Delphi panel to validate or adapt the model’s structure and all the parameters required to populate the budget impact model.

# **Supplementary Material Table S2.** Total drug cost estimation in active treatment period and post-active treatment period. Values expressed in $ dollars 2020.

| **Treatment** | **Dosing schedule** | **Ex-factory price per pack** | **N of administrations** | **Ex-factory price per administration** | **Number of administrations per cycle** | **Dose intensity*** | **Drug cost per patient per cycle** | **Mean treatment duration (cycles)** | **Total drug cost per patient in active treatment period** | **Post active treatment duration (cycles)** | **Total drug cost per patient in post- active treatment period** | **Total drug cost per patient** |
| --- | --- | --- | --- | --- | --- | --- | --- | --- | --- | --- | --- | --- |
|  |  | A | B | C=(A*B) | D | E | F=C*D*E | G | H=F*G | I | J= I* Px BSC** | K=H+J |
| **Venetoclax + azacitidine** |  |  |  |  |  |  | **$18.628** |  | **$184.766** | **2,07** | **$1.445** | **$186.207** |
| Venetoclax [First cycle: treatment initiation] | 100 mg, 200 mg, 400 mg on Days 1, 2, 3 | $46 | 3 | $138 | 3 | 0,27 | $113 | 1 | $88 |  |  |  |
| Venetoclax [First cycle: post treatment initiation] | 400 mg daily on Days 4-28 |  | 4 | $184 | 25 | 0,36 | $1.674 | 1 | $1.674 |  |  |  |
| Venetoclax [Subsequent cycles] | 400 mg daily for 28 days |  | 4 | $184 | 28 | 0,36 | $1.875 | 10 | $18.706 |  |  |  |
| Azacitidine | 75 mg/m² daily for 7 days | $1.069 | 2 | $2.138 | 7 | 1,00 | $14.966 | 11 | $164.298 |  |  |  |
| **Venetoclax + LDAC** |  |  |  |  |  |  | **$4.635** |  | **$16.824** | **5,99** | **$4.175** | **$20.999** |
| Venetoclax [First cycle: treatment initiation] | 100 mg, 200 mg, 400 mg, 600 mg on Days 1, 2, 3, 4 | $46 | 4 | $184 | 4 | 0,29 | $211 | 1 | $172 |  |  |  |
| Venetoclax [First cycle: post treatment initiation] | 600 mg daily on Days 5-28 |  | 6 | $276 | 24 | 0,30 | $2.016 | 1 | $2.016 |  |  |  |
| Venetoclax [Subsequent cycles] | 600 mg daily for 28 days |  | 6 | $276 | 28 | 0,30 | $2.352 | 6 | $14.244 |  |  |  |
| LDAC | 20 mg/m² daily for 10 days | $6 | 1 | $6 | 10 | 1,00 | $56 | 7 | $392 |  |  |  |
| **Venetoclax + decitabine** |  |  |  |  |  |  | **$9.914** |  | **$88.789** | **2,11** | **$1.473** | **$90.262** |
| Venetoclax [First cycle: treatment initiation] | 100 mg, 200 mg, 400 mg on Days 1, 2, 3 | $46 | 3 | $138 | 3 | 0,27 | $113 | 1 | $113 |  |  |  |
| Venetoclax [First cycle: post treatment initiation] | 400 mg daily on Days 4-28 |  | 4 | $184 | 25 | 0,36 | $1.674 | 1 | $1.674 |  |  |  |
| Venetoclax [Subsequent cycles] | 400 mg daily for 28 days |  | 4 | $184 | 28 | 0,36 | $1.875 | 10 | $18.639 |  |  |  |
| Decitabine | 20 mg/m² daily for 5 days | $1.251 | 1 | $1.251 | 5 | 1,00 | $6.253 | 11 | $68.402 |  |  |  |
| **Azacitidine** | 75 mg/m² daily for 7 days | **$1.069** | **2** | **$2.138** | **7** | **1,00** | **$14.966** | **9** | **$131.701** | **4,24** | **$2.961** | **$134.662** |
| **LDAC** | 20 mg/m² daily for 10 days | **$6** | **1** | **$6** | **10** | **1,00** | **$56** | **4** | **$209** | **9,28** | **$6.473** | **$6.682** |
| **Decitabine** | 20 mg/m² daily for 5 days | **$1.251** | **1** | **$1.251** | **5** | **1,00** | **$6.253** | **7** | **$43.142** | **6,14** | **$4.288** | **$47.430** |

* Dose intensity is an adjustment made to the amount of VTX administration for the use of antifungals used as prophylaxis in AML, based on opinion of Delphi Panel

** The drug cost of BSC per cycle was estimated at $698 through the micro-costing approach.
