# Supplementary material for: Budget impact analysis of venetoclax for the management of acute myeloid leukemia from the perspective of the social security and the private sector in Argentina
Source: PLoS One. 2024 Jan 4;19(1):e0295798. doi: 10.1371/journal.pone.0295798 (PMC10766175; doi:10.1371/journal.pone.0295798)
Supplement: S5 Table — (DOCX) [file pone.0295798.s005.docx]

*PLOS ONE*

**Budget impact analysis of venetoclax for the management of acute myeloid leukemia from the perspective of the social security and the private sector in Argentina**

Alfredo Palacios,^1,2,3^ Natalia Espinola,^1^ Juan Martin Gonzalez,^1^ Carlos Rojas-Roque,^1,3^ Maria Marta Rivas,^4^ Diego Kanevski,^5^ Pierre Morisset,^5^ Federico Augustovski,^1^ Andres Pichon-Riviere,^1^ Ariel Bardach^1^

^1^ Department of Health Technology Assessment and Health Economics, Institute for Clinical Effectiveness and Health Policy (IECS), Buenos Aires, Argentina

^2^ Department of Economics, Universidad de Buenos Aires, Buenos Aires, Argentina

^3^ Centre for Health Economics (CHE), University of York, York, UK

^4^ Hospital Universitario Austral, Buenos Aires, Argentina

^5^ AbbVie Argentina, Ing. Enrique Butty 240, C1106 CABA, Argentina

**Corresponding author**

Alfredo Palacios, MSc

Department of Health Technology Assessment and Health Economics, Institute for Clinical Effectiveness and Health Policy (IECS), Buenos Aires, Argentina

Department of Economics, Universidad de Buenos Aires, Buenos Aires, Argentina

Centre for Health Economics (CHE), University of York, York, UK

Email: alfredo.palacios@york.ac.uk

ORCID: 0000-0001-7684-0880

**Declarations**

**Conflicts of interest/Competing interests**

I have read the journal's policy and the authors of this manuscript have the following competing interests. Alfredo Palacios, Natalia Espinola, Juan Martin González, Carlos Rojas-Roque, Andrés Pichon-Riviere, Federico Augustovski and Ariel Bardach declare that they have no conflicts of interest. Diego Kanevsky and Pierre Morisset are employees of Abbvie and may own Abbvie stocks. Maria Marta Rivas has received speaker fees from Abbvie. This does not alter our adherence to PLOS ONE policies on sharing data and materials.

**Data Accessibility Statement**

All parameters used to populate the budget impact model are provided within the main manuscript and its supplementary material. The budget impact model itself will be made available upon reasonable request.

**Consent for publication**.

Not applicable.

**Acknowledgements.**

The authors wish to thank Isolda Fernandez, Mariela Gómez, Hernán Dick, Laura Fischman and Irene Rey, who participated in the modified Delphi panel to validate or adapt the model’s structure and all the parameters required to populate the budget impact model.

# **Supplementary Material Table S7.** Distribution of the target population for the current and projected scenario.

| **Scenario** | **Regimen** | **Year 1** | **Year 2** | **Year 3** |
| --- | --- | --- | --- | --- |
| Current scenario |  |  |  |  |
|  | AZA | 75 | 75 | 75 |
|  | DE | 15 | 15 | 15 |
|  | LDC | 15 | 15 | 15 |
|  | BSC | 24 | 24 | 24 |
|  | Total | 129 | 129 | 129 |
| Projected scenario |  |  |  |  |
|  | VEN + AZA | 26 | 45 | 49 |
|  | VEN + DE | 12 | 12 | 12 |
|  | VEN + LDC | 19 | 19 | 19 |
|  | AZA | 29 | 18 | 16 |
|  | DE | 11 | 8 | 8 |
|  | LDC | 13 | 10 | 8 |
|  | BSC | 19 | 17 | 17 |
|  | Total | 129 | 129 | 129 |

**Abbreviations.** BSC, best supportive care; AZA, azacitidine; DB, decitabine; LDC, low-dose cytarabine; VEN, venetoclax.
