# Supplementary material for: Budget impact analysis of venetoclax for the management of acute myeloid leukemia from the perspective of the social security and the private sector in Argentina
Source: PLoS One. 2024 Jan 4;19(1):e0295798. doi: 10.1371/journal.pone.0295798 (PMC10766175; doi:10.1371/journal.pone.0295798)
Supplement: S1 File — (DOCX) [file pone.0295798.s006.docx]

*PLOS ONE*

**Budget impact analysis of venetoclax for the management of acute myeloid leukemia from the perspective of the social security and the private sector in Argentina**

Alfredo Palacios,^1,2,3^ Natalia Espinola,^1^ Juan Martin Gonzalez,^1^ Carlos Rojas-Roque,^1,3^ Maria Marta Rivas,^4^ Diego Kanevski,^5^ Pierre Morisset,^5^ Federico Augustovski,^1^ Andres Pichon-Riviere,^1^ Ariel Bardach^1^

^1^ Department of Health Technology Assessment and Health Economics, Institute for Clinical Effectiveness and Health Policy (IECS), Buenos Aires, Argentina

^2^ Department of Economics, Universidad de Buenos Aires, Buenos Aires, Argentina

^3^ Centre for Health Economics (CHE), University of York, York, UK

^4^ Hospital Universitario Austral, Buenos Aires, Argentina

^5^ AbbVie Argentina, Ing. Enrique Butty 240, C1106 CABA, Argentina

**Corresponding author**

Alfredo Palacios, MSc

Department of Health Technology Assessment and Health Economics, Institute for Clinical Effectiveness and Health Policy (IECS), Buenos Aires, Argentina

Department of Economics, Universidad de Buenos Aires, Buenos Aires, Argentina

Centre for Health Economics (CHE), University of York, York, UK

Email: alfredo.palacios@york.ac.uk

ORCID: 0000-0001-7684-0880

**Declarations**

**Conflicts of interest/Competing interests**

I have read the journal's policy and the authors of this manuscript have the following competing interests. Alfredo Palacios, Natalia Espinola, Juan Martin González, Carlos Rojas-Roque, Andrés Pichon-Riviere, Federico Augustovski and Ariel Bardach declare that they have no conflicts of interest. Diego Kanevsky and Pierre Morisset are employees of Abbvie and may own Abbvie stocks. Maria Marta Rivas has received speaker fees from Abbvie. This does not alter our adherence to PLOS ONE policies on sharing data and materials.

**Data Accessibility Statement**

All parameters used to populate the budget impact model are provided within the main manuscript and its supplementary material. The budget impact model itself will be made available upon reasonable request.

**Consent for publication**.

Not applicable.

**Acknowledgements.**

The authors wish to thank Isolda Fernandez, Mariela Gómez, Hernán Dick, Laura Fischman and Irene Rey, who participated in the modified Delphi panel to validate or adapt the model’s structure and all the parameters required to populate the budget impact model.

# **Supplementary Material S5.** Questionnaire (in Spanish) for the modified Delphi Panel

**Análisis de impacto presupuestario de Venetoclax en combinación con Azacitidina, Decitabina o dosis bajas de Citarabina en Leucemia Mieloide Aguda de reciente diagnóstico en pacientes mayores de 65 años de edad no candidatos a inducción plena, desde la perspectiva de la Seguridad Social y el Sector Privado de salud en Argentina**

***Ejercicio de validación y consenso de expertos***

Iniciales: _______ Fecha:

**Palabras de bienvenida**

Estimado participante, estamos encantados de recibirlo en el Instituto de Efectividad Clínica y Sanitaria (IECS). La presente actividad tiene como objetivo relevar y/o validar datos epidemiológicos y del manejo clínico de la Leucemia Mieloide Aguda (LMA) de reciente diagnóstico para pacientes mayores de 65 años no candidatos a inducción plena.

Este proyecto de investigación es llevado a cabo por el IECS, financiado con un subsidio de investigación independiente de la compañía farmacéutica Abbvie. Las preguntas que usted y el resto de los expertos respondan se refieren a información que no ha podido ser caracterizada adecuadamente en la búsqueda y revisión de la literatura científica llevada a cabo por nuestro equipo de investigación. Por lo tanto, apelamos a su conocimiento y experiencia en el tema para ayudar a dar respuesta a estas incógnitas, necesarias para llevar adelante nuestro estudio.

Es importante remarcar que toda la información provista por usted será tratada en forma totalmente confidencial.

**Sobre las actividades y agenda**

La actividad durará como máximo 4 hs. Inicialmente, se presentará el proyecto de investigación y se brindarán las instrucciones sobre cómo llenar el cuestionario. Luego, se realizará la primera ronda de llenado del cuestionario, que durará como máximo 75 minutos. En esta etapa usted debe completar el cuestionario utilizando un bolígrafo de color azul. Los cuestionarios respondidos deberán ser entregados a los miembros del equipo IECS a medida que se vayan completando, para favorecer la carga y procesamiento de los datos.

Posteriormente, tendremos una pausa de 20 minutos, durante la cual el equipo IECS procesará las respuestas obtenidas y generará estadísticas agregadas a nivel del panel de expertos (promedios, valores mínimos y máximos, etc.). Al retomar las actividades, se devolverán los cuestionarios y se discutirán los resultados obtenidos.

Finalmente, se procederá a una segunda vuelta de respuestas del cuestionario, que se realizará sobre el cuestionario original, esta vez utilizando un bolígrafo de color rojo. Esto es para que, en el caso que usted así lo considere, pueda corregir las respuestas brindadas en la primera ronda. Finalmente, se deberá entregar el cuestionario modificado a los miembros del equipo de IECS.

**Consideraciones sobre la información que solicitamos**

- ***Perspectiva*:** Al responder las preguntas usted debe pensar en un escenario convencional y promedio de Argentina, que incluya la variabilidad esperada dentro del subsector de la seguridad social (obras sociales) y el subsector privado de salud.
- ***Población y definición del caso sobre el que realizaremos las preguntas*:** La población son pacientes adultos mayores de 65 años de edad con Leucemia Mieloide Aguda (LMA) recientemente diagnosticada que no son candidatos a recibir tratamiento quimioterápico de inducción plena.

**Tipo de información solicitada**

La información que le solicitaremos es sobre porcentajes, cantidades temporales (cantidad de días, ciclos, meses, etc.), y cantidades de recursos sanitarios (número de consultas, número de días de internación, de estudios de laboratorio, etc.). A su vez, en algunos casos le pediremos que responda por el orden en la secuencia de tratamientos (posición esperada de cada tratamiento disponible en Argentina), su percepción sobre la participación de cada tratamiento en el mercado argentino de la patología (en términos porcentuales).

*A su vez, le consultaremos sobre la utilización esperada de recursos sanitarios durante los tratamientos. En particular, le preguntaremos sobre la siguiente información:*

*Cantidades del recurso sanitario (valores base, rango del intervalo de confianza al 95%):* En este caso se le solicita indicar, de acuerdo a lo especificado por cada pregunta, la cantidad de unidades del recurso sanitario que espera que un paciente utilice por el tiempo indicado (por ejemplo, cantidad de días de internación en 1 mes). En todos los casos, debe indicar un valor promedio y un mínimo y máximo del intervalo de confianza del 95%, que indica que la media de la *población* estará con un 95% de probabilidades dentro de ese rango.

*Porcentaje del total de pacientes:* Usted debe contestar sobre el porcentaje de pacientes mayores de 65 años de edad con LMA no candidatos a tratamiento quimioterápico de inducción plena en Argentina que espera utilicen al menos una vez el recurso sanitario analizado. Este porcentaje de utilización será un valor comprendido entre 0% y 100%. Por ejemplo, si en el seguimiento de 100 pacientes durante el tratamiento se espera que la mitad de los mismos realice una determinación de su valor de glucemia, debería indicar que el 50% de los pacientes utiliza el recurso “determinación del valor de glucemia”.

**Instructivo**

A continuación, se presenta un ejemplo de pregunta de este cuestionario y sus correspondientes campos para el llenado.


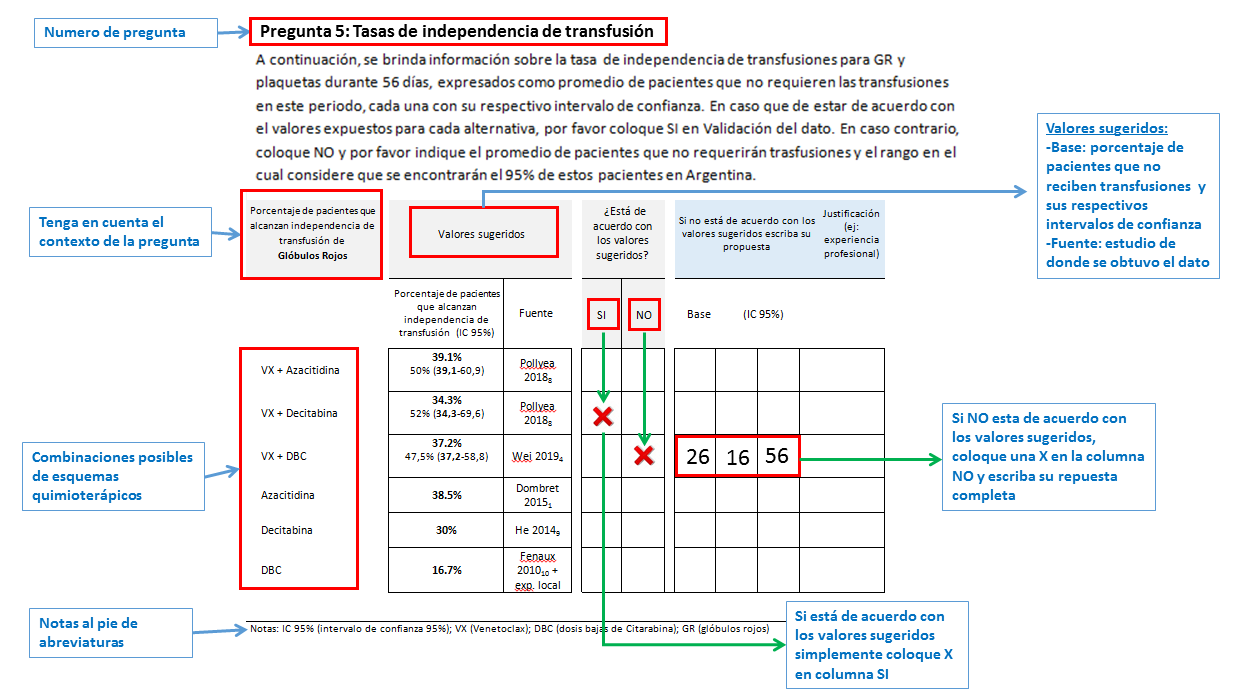


Nota: no complete los casilleros que se encuentran coloreados en gris.

**CUESTIONARIO**

**Aspectos epidemiológicos**

1. **Pacientes mayores de 65 años de edad con LMA no candidatos a inducción quimioterápica plena**

La información sobre el porcentaje de pacientes con diagnóstico de LMA mayores de 65 años de edad que NO son candidatos a inducción plena, y que reciben esquemas quimioterápicos con hipometilantes o dosis bajas de Citarabina (DBC) en monoterapia y en combinación con Venetoclax, fue obtenida a partir de estudios locales e internacionales:

- El ensayo clínico aleatorizado multicéntrico en fase III realizado por Dombret y cols. (2015)_1_, incluyó pacientes mayores de 65 años de edad, con diagnóstico reciente de LMA que no hubieran recibido tratamiento quimioterápico previamente. Del total de pacientes incluidos (n=488), el 82% (n=399) fueron considerados NO candidatos a inducción plena, por lo que recibieron esquemas quimioterápicos alternativos.

- Con respecto a la disponibilidad de datos para la Argentina, se identificó el estudio desarrollado por Mela Osorio y cols. (2019)_2_, que evaluó el impacto de hipometilantes y quimioterapia de inducción intensiva en pacientes con LMA mayores de 65 años. Este estudio multicéntrico y retrospectivo surge de un sub-análisis de la base de datos de la Subcomisión de Leucemias Agudas de la Sociedad Argentina de Hematología. De un total de 728 pacientes, se seleccionaron 145 pacientes mayores de 65 años con LMA (20%). De éstos, el 60% (n=87) NO fueron candidatos a terapia de inducción intensiva.

- Por favor, en base a su experiencia y la información arriba presentada, indique el ***porcentaje de pacientes*** ***mayores de 65 a*ños** con diagnóstico reciente de ***LMA*** ***que usted considera que NO serán candidatos a recibir tratamiento quimioterápico de inducción plena***, y el rango en el cual considere se encontrarán el 95% de estos pacientes en Argentina.

|  |  |  | Valores sugeridos por literatura médica |  | Su respuesta | | | Justificación (ej: experiencia profesional) |
| --- | --- | --- | --- | --- | --- | --- | --- | --- |
|  |  |  |  |  | Base | (IC 95%) | |  |
|  |  |  |  |  |  |  |  |  |
|  | Porcentaje de pacientes mayores de 65 años con LMA no candidatos a inducción plena |  | 82%_1_ |  |  |  |  |  |
|  |  |  | 60%_2_ |  |  |  |  |  |
|  |  |  |  |  |  |  |  |  |

Notas: IC 95% (intervalo de confianza 95%)

1. Dombret y cols. 2015
2. Mela Osorio y cols. 2019

**Aspectos clínicos**

1. **Duración del tratamiento activo**

La duración total de los tratamientos quimioterápicos alternativos varía según el tipo de respuesta observada en los pacientes.

Se considera periodo de ***tratamiento activo*** al periodo comprendido entre el inicio de la administración de las terapias bajo estudio hasta su finalización, según la respuesta clínica observada. Cada ciclo de tratamiento dura 28 días.

La publicación de Di Nardo y cols. (2018)_3_ reporta una mediana de duración de 5 ciclos (rango 1-25) para los tratamientos con Venetoclax en combinación con hipometilantes, mientras que el estudio de Wei y cols. (2014)_4_ reporta una mediana de duración de 4,2 ciclos (rango 0,2-29) para el tratamiento de Venetoclax combinado con Dosis Bajas con Citarabina (DBC). Sin embargo, debido a que estos datos provienen de los estudios iniciales de Venetoclax, y no contemplan la totalidad de la información que se encuentra actualmente disponible, se procedió a la utilización de los datos provistos por el laboratorio Abbvie a partir del estudio M14-358/387_5_, el cual cuenta con un mayor número de pacientes y sus datos son presentados con un mayor grado de detalle.

- A continuación, se presenta la información sobre la duración promedio de los tratamientos con las alternativas quimioterápicas consideradas y sus respectivas fuentes bibliográficas. La duración promedio de cada tratamiento es expresada como el número promedio de ciclos (de 28 días), con su respectivo rango de duración.

En caso de estar de acuerdo con los valores sugeridos para cada alternativa de tratamiento, por favor coloque **SI** en el casillero correspondiente. En caso contrario, coloque **NO** y por favor indique el número promedio de ciclos de duración del/los tratamientos/s, y el rango de duración en el que considere se encontrarán el 95% de estos pacientes en Argentina.

| Duración del tratamiento activo |  | Valores sugeridos | |  | ¿Está de acuerdo con los valores sugeridos? | |  | Si no está de acuerdo con los valores sugeridos escriba su propuesta | | | Justificación |
| --- | --- | --- | --- | --- | --- | --- | --- | --- | --- | --- | --- |
|  |  |  |  |  |  |  |  |  |  |  |  |
|  |  |  |  |  |  |  |  |  |  |  |  |
|  |  | Media de duración -en ciclos- y (rango) |  |  |  |  |  |  |  | |  |
|  |  |  | Fuente |  | **Si** | **No** |  | Base | (IC 95%) | |  |
|  |  |  |  |  |  |  |  |  |  | |  |
| VX + azacitidina |  | **7,51** | Estudio M14-358_5_ |  |  |  |  |  |  |  |  |
|  |  | (0,11; 34,64) |  |  |  |  |  |  |  |  |  |
| VX + Decitabina |  | **10,94** | Estudio M14-358_5_ |  |  |  |  |  |  |  |  |
|  |  | (0,54; 38,96) |  |  |  |  |  |  |  |  |  |
| VX + DBC |  | **7,63** | Estudio M14-387_5_ |  |  |  |  |  |  |  |  |
|  |  | (0,25; 31,75) |  |  |  |  |  |  |  |  |  |
| Azacitidina |  | **8,8** | AZA NICE appraisal 2017_6_ |  |  |  |  |  |  |  |  |
| Decitabina |  | **6,9** | Kantarjian 2012_7_ |  |  |  |  |  |  |  |  |
|  |  |  |  |  |  |  |  |  |  |  |  |
| DBC |  | **3,76** | Kantarjian 2012_7_ |  |  |  |  |  |  |  |  |

Notas: IC 95% (intervalo de confianza 95%); VX (Venetoclax); DBC (dosis bajas de Citarabina)

1. **Duración de hospitalizaciones en pacientes que presenten o no progresión de la enfermedad**

Se asume que los pacientes que NO alcanzan la RC/RCi (Remisión Completa/Remisión Completa con recuperación incompleta del recuento sanguíneo) presentan progresión de su enfermedad. La progresión de la enfermedad implica hospitalizaciones debido a complicaciones asociadas a la patología de base. Los días de hospitalización que requieran estos pacientes pueden variar dependiendo de si los pacientes aún se encuentran recibiendo *tratamiento activo*, es decir, durante el período de administración del tratamiento propiamente dicho, o la progresión se da luego de los ciclos del tratamiento *(post tratamiento activo)*. En este período con progresión, luego de finalizados los ciclos terapéuticos o de post tratamiento activo, se asume que los pacientes reciben el mejor tratamiento de soporte (*best supportive care*).

- Por favor, indique la cantidad promedio de días de hospitalización por ciclo (de 28 días) que requiere un paciente en cada situación presentada y el rango en el cual considere se encontrarán el 95% de estos pacientes en Argentina.

|  |  | Valores sugeridos* | |  | Su respuesta | | | Justificación (ej: experiencia profesional) |
| --- | --- | --- | --- | --- | --- | --- | --- | --- |
|  |  | Ciclos 1 y 2 | Ciclos subsiguientes |  | Base | (IC 95%) | |  |
|  |  |  |  |  |  |  |  |  |
| Duración promedio de la hospitalización (en días)  por ciclo, para pacientes que SI alcanzan  la RC/RCi en Tratamiento Activo |  | 20 | 2 |  |  |  |  |  |
|  |  |  |  |  |  |  |  |  |
|  |  |  |  |  |  |  |  |  |
| Duración promedio de la hospitalización (en días)  por ciclo para pacientes que NO  alcanzan la RC/RCi en Tratamiento Activo |  | 20 | 15 |  |  |  |  |  |
|  |  |  |  |  |  |  |  |  |
|  |  |  |  |  |  |  |  |  |
| Duración promedio de la hospitalización (en días)  Por ciclo para pacientes que NO alcanzan  la RC/Rci en Post Tratamiento Activo  (*best supportive care*) |  | NA | 20 |  |  |  |  |  |
|  |  |  |  |  |  |  |  |  |

Notas: IC 95% (intervalo de confianza 95%); RC (remisión completa); RCi (remisión completa con recuperación incompleta del recuento sanguíneo); NA (No Aplica).

*Fuente: Opinión de experta local

1. **Distribución del uso de antifúngicos utilizados como profilaxis en LMA**

Se tiene conocimiento de que la mayoría de los pacientes que reciben tratamiento para LMA reciben profilaxis con antifúngicos a fin de evitar las infecciones invasivas por estos microorganismos. La elección del antifúngico no se encuentra definida en las guías de práctica clínica debido a eficacias similares, por lo que dicha elección suele estar sujeta a condiciones de disponibilidad, costos, posología y práctica habitual de cada centro de salud.

- A continuación, se solicita que indique el porcentaje de pacientes mayores de 65 años con LMA no candidatos a recibir inducción intensiva que son pasibles de tratamiento profiláctico antifúngico en los subsectores de la seguridad social y privado de Argentina, y el rango en el cual considere que se encontrarán el 95% de estos pacientes.

Ejemplo: de cada 100 pacientes candidatos a recibir tratamiento profiláctico, 10 de ellos reciben tratamiento con posaconazol (10%).

|  | |  | Valores sugeridos* |  | Su respuesta | | | Justificación (ej: experiencia profesional) |
| --- | --- | --- | --- | --- | --- | --- | --- | --- |
|  |  |  |  |  | Base | (IC 95%) | |  |
|  | |  |  |  |  |  |  |  |
|  | Porcentaje de pacientes candidatos recibir tratamiento profiláctico con **posaconazol** |  | 10% |  |  |  |  |  |
|  | Porcentaje de pacientes con profilaxis con inhibidores fuertes (**voriconazol, itraconazol**) |  | 60% |  |  |  |  |  |
|  | Porcentaje de pacientes con profilaxis con inhibidores moderados (**fluconazol**) |  | 20% |  |  |  |  |  |
|  | Porcentaje de pacientes que **no reciben** profilaxis antifúngica |  | 10% |  |  |  |  |  |
|  | Total |  | 100% |  |  |  |  |  |
|  |  |  |  |  |  |  |  |  |

Notas: IC 95% (intervalo de confianza 95%)

*Fuente: Opinión de experta local

1. **Tasas de independencia de transfusión**

Como medida adicional de eficacia de los tratamientos quimioterápicos alternativos, se considera la independencia de transfusiones de glóbulos rojos (GR) y plaquetas durante 2 ciclos (equivalentes a 56 días).

Debido a que los estudios de Venetoclax actualmente disponibles fueron catalogados como de baja calidad metodológica (ya que se trata de estudios observacionales no controlados y por ende sin asignación ciega y aleatorizada de ramas de tratamiento), se consideraron los límites inferiores (**resaltados en negrita**) de los intervalos de confianza reportados por los estudios actualmente disponibles. Esta estrategia representaría una aproximación “conservadora” respecto a la eficacia observada en los estudios de Venetoclax actualmente disponibles.

- A continuación, se brinda información sobre la tasa de independencia de transfusiones para GR y plaquetas durante 56 días, expresados como promedio de pacientes que NO requieren las transfusiones en ese periodo, con su respectivo intervalo de confianza. En caso que de estar de acuerdo con los valores sugeridos para cada alternativa de tratamiento, por favor coloque SI. En caso contrario, coloque NO y por favor indique (de acuerdo a su conocimiento y/o experiencia) el promedio de pacientes que NO requerirán trasfusiones y el rango en el cual considere que se encontrarán el 95% de estos pacientes en Argentina.

| Porcentaje de pacientes que alcanzan independencia de transfusión de **Glóbulos Rojos** |  | Valores sugeridos | |  | ¿Está de acuerdo con los valores sugeridos? | |  | Si no está de acuerdo con los valores sugeridos escriba su propuesta | | | Justificación |
| --- | --- | --- | --- | --- | --- | --- | --- | --- | --- | --- | --- |
|  |  |  |  |  |  |  |  |  |  |  |  |
|  |  |  |  |  |  |  |  |  |  |  |  |
|  |  | Porcentaje de pacientes que alcanzan independencia de transfusión (IC 95%) | Fuente |  | **Si** | **No** |  | Base | (IC 95%) | |  |
|  |  |  |  |  |  |  |  |  |  |  |  |
| VX + Azacitidina |  | **39.1%** | Pollyea 2018_8_ |  |  |  |  |  |  |  |  |
|  |  | 50% (**39,1**-60,9) |  |  |  |  |  |  |  |  |  |
| VX + Decitabina |  | 34.3% | Pollyea 2018_8_ |  |  |  |  |  |  |  |  |
|  |  | 52% (**34,3**-69,6) |  |  |  |  |  |  |  |  |  |
| VX + DBC |  | **37.2%** | Wei 2019_4_ |  |  |  |  |  |  |  |  |
|  |  | 47,5% (**37,2**-58,8) |  |  |  |  |  |  |  |  |  |
| Azacitidina |  | **38.5%** | Dombret 2015_1_ |  |  |  |  |  |  |  |  |
| Decitabina |  | **30%** | He 2014_9_ |  |  |  |  |  |  |  |  |
|  |  |  |  |  |  |  |  |  |  |  |  |
| DBC |  | **16.7%** | Fenaux 2010_10_ + exp. local |  |  |  |  |  |  |  |  |

Notas: IC 95% (intervalo de confianza 95%); VX (Venetoclax); DBC (dosis bajas de Citarabina); GR (glóbulos rojos)

| Porcentaje de pacientes que alcanzan independencia de transfusión de **Plaquetas** |  | Valores sugeridos | |  | ¿Está de acuerdo con los valores sugeridos? | |  | Si no está de acuerdo con los valores sugeridos escriba su propuesta | | | Justificación |
| --- | --- | --- | --- | --- | --- | --- | --- | --- | --- | --- | --- |
|  |  |  |  |  |  |  |  |  |  |  |  |
|  |  |  |  |  |  |  |  |  |  |  |  |
|  |  | Porcentaje de pacientes que alcanzan independencia de transfusión (IC 95%) | Fuente |  | **Si** | **No** |  | Base | (IC 95%) | |  |
|  |  |  |  |  |  |  |  |  |  |  |  |
| VX + Azacitidina |  | **47.2%** | Pollyea 2018_8_ |  |  |  |  |  |  |  |  |
|  |  | 58% (**47,2**-68,7) |  |  |  |  |  |  |  |  |  |
| VX + Decitabina |  | **42.85%** | Pollyea 2018_8_ |  |  |  |  |  |  |  |  |
|  |  | 60% (**42,8**-77,2) |  |  |  |  |  |  |  |  |  |
| VX + DBC |  | **49.4%** | Wei 2019_4_ |  |  |  |  |  |  |  |  |
|  |  | 59,76% (49,4-70,6) |  |  |  |  |  |  |  |  |  |
| Azacitidina |  | **40.6%** | Dombret 2015_1_ |  |  |  |  |  |  |  |  |
| Decitabina |  | **48%** | He 2014_9_ |  |  |  |  |  |  |  |  |
|  |  |  |  |  |  |  |  |  |  |  |  |
| DBC |  | **20%** | Fenaux 2010_10_ + exp. local |  |  |  |  |  |  |  |  |

Notas: IC 95% (intervalo de confianza 95%); VX (Venetoclax); DBC (dosis bajas de Citarabina)

**Aspectos relacionados a cuotas de mercado de drogas quimioterápicas**

1. **Cuotas de mercado de las drogas quimioterápicas actualmente disponibles en Argentina**

A continuación, se presentan los posibles esquemas quimioterápicos **actualmente disponibles** en Argentina para pacientes con LMA mayores de 65 años de edad no candidatos a tratamiento quimioterápico de inducción intensiva.

- Por favor, indique el porcentaje (%) en el que usted considera se utiliza cada régimen de medicamentos en el conjunto del subsector de la seguridad social y el sector privado de salud de Argentina:

|  | |  | Valores sugeridos* |  | Su respuesta | | | Justificación (ej: experiencia profesional) |
| --- | --- | --- | --- | --- | --- | --- | --- | --- |
|  | |  |  |  | Base | (IC 95%) | |  |
|  | |  |  |  |  |  |  |  |
|  | Azacitidina |  | 43% |  |  |  |  |  |
|  | Dosis baja de citarabina |  | 18% |  |  |  |  |  |
|  | Decitabina |  | 29% |  |  |  |  |  |
|  | *Best supportive care*** |  | 10% |  |  |  |  |  |
|  | Total |  | 100% |  |  |  |  |  |

*Fuente: Estimaciones provistas por el laboratorio Abbvie.

**Tratamiento de soporte

1. **Proyección de las cuotas de mercado de las drogas quimioterápicas ante la incorporación de Venetoclax en Argentina**

A continuación, se presenta un posible **escenario de proyección a 3 años del uso efectivo de Venetoclax** como complemento del esquema de tratamiento actual en pacientes adultos mayores de 65 años con diagnóstico de LMA no candidatos a inducción plena.

- Por favor, indique el porcentaje del total de pacientes mayores de 65 años con LMA no candidatos a inducción plena que usted considere recibirán en forma efectiva Venetoclax si tal droga se incorporara a la cobertura brindada por el conjunto del subsector de la seguridad social y privado de salud de Argentina. Tenga en cuenta en su estimación las posibles barreras (administrativas, o de otra índole) podrían existir en dichos sectores para el uso efectivo de la droga.

|  | | |  | Valor sugerido* | | |  | Su respuesta | | | | |
| --- | --- | --- | --- | --- | --- | --- | --- | --- | --- | --- | --- | --- |
|  | | |  | Año 1 | Año 2 | Año 3 |  | Año 1 | Año 2 | | Año 3 | |
|  | | |  |  |  |  |  |  |  | |  | |
|  | VX + Azacitidina |  | | 20% | 29% | 29% |  |  | |  | |  |
|  | VX + DBC |  | | 8% | 12% | 13% |  |  | |  | |  |
|  | VX + Decitabina |  | | 13% | 19% | 19% |  |  | |  | |  |
|  | Azacitidina |  | | 24% | 14% | 13% |  |  | |  | |  |
|  | DBC |  | | 9% | 6% | 5% |  |  | |  | |  |
|  | Decitabina |  | | 16% | 11% | 10% |  |  | |  | |  |
|  | *Best supportive care* |  | | 10% | 10% | 10% |  |  | |  | |  |
|  | **El Total debe ser 100%** |  | | **100%** | **100%** | **100%** |  |  | |  | |  |

Notas: IC 95% (intervalo de confianza 95%); DBC (dosis bajas de Citarabina)

*Fuente: Estimaciones provistas por el laboratorio Abbvie.

**Aspectos relacionados al uso de recursos sanitarios**

1. **Uso de recursos sanitarios en el cuidado de rutina durante el tratamiento, anual**

A continuación, se presentan los recursos sanitarios, sus tasas de uso (% del total de pacientes que utilizan dicho recurso) y sus cantidades, utilizados en el cuidado de rutina durante el tratamiento para pacientes adultos con LMA en un año.

- Por favor, realice su estimación de recursos sanitarios utilizados en un año (identificación de recursos sanitarios, tasas de uso (%) y cantidades) recurriendo a su experiencia profesional, siempre intentando reflejar la práctica habitual y esperable en los subsectores de la seguridad social y privado de salud de Argentina.

Ejemplo: de 100 pacientes que se encuentran en tratamiento, a 100 (% del total de pacientes) se le pide 12 paneles químicos por año.

| **Uso de recursos sanitarios en monitoreo anual** | |  | Valores sugeridos* | |  | Su respuesta | |
| --- | --- | --- | --- | --- | --- | --- | --- |
|  |  |  | % del  total de pacientes | Cantidad anual |  | % del  total de pacientes | Cantidad anual |
|  |  |  |  |  |  |  |  |
|  |  |  |  |  |  |  |  |
|  | Conteo de Sangre Completo (Hemograma) |  | 100% | 12 |  |  |  |
|  | Panel químico (función renal, test clearance creatinina, test bilirrubina, hepatograma, ionograma, calcemia, fosfatemia, ácido úrico sérico, proteínas totales, glucemia, albumina sérica, LDH, cloro sérico, bicarbonato) |  | 100% | 12 |  |  |  |
|  | Biopsia médula ósea |  | 100% | 1 |  |  |  |
|  | Aspiración de médula ósea |  | 100% | 2** |  |  |  |
|  | Otro 1: |  |  |  |  |  |  |
|  | Otro 2: |  |  |  |  |  |  |
|  | Otro 3: |  |  |  |  |  |  |
|  |  |  |  |  |  |  |  |

*Fuente: Opinión de experta local

** 1 para diagnóstico y 1 para evaluar respuesta

1. **Cantidad de transfusiones por ciclo (28 días)**

Se estima que los pacientes se transfunden con glóbulos rojos los dos o tres primeros meses de tratamiento, con una frecuencia de 3 transfusiones por ciclo, y si el paciente responde a la terapia, deja de recibirlas. En el caso de transfusiones con plaquetas, se estima que los pacientes las reciben durante unos seis meses o lo que viva el paciente, con una frecuencia por ciclo de 5 transfusiones. La cantidad promedio de transfusiones fue obtenida por medio de opinión de una experta local.

- Por favor, realice su estimación de la cantidad promedio de transfusiones de glóbulos rojos y plaquetas realizados por ciclo (28 días) en periodo de tratamiento activo para pacientes con LMA, recurriendo a su experiencia profesional, siempre intentando reflejar la práctica habitual y esperable de los subsectores de la seguridad social y privado de salud de Argentina.

| **Cantidad de transfusiones** | |  | Valores sugeridos* | |  | Su respuesta | |
| --- | --- | --- | --- | --- | --- | --- | --- |
|  |  |  | % del  total de pacientes | Cantidad por ciclo |  | % del  total de pacientes | Cantidad por ciclo |
|  |  |  |  |  |  |  |  |
|  |  |  |  |  |  |  |  |
|  | Transfusión de Glóbulos Rojos por ciclo |  | 100% | 3 |  |  |  |
|  | Transfusión de Plaquetas por ciclo |  | 100% | 5 |  |  |  |
|  |  |  |  |  |  |  |  |

*Fuente: Opinión de experta local

***¡Muchas gracias!***

**Referencias**

1. Dombret, H. *et al.* International phase 3 study of azacitidine vs conventional care regimens in older patients with newly diagnosed AML with >30% blasts. *Blood* **126**, 291–299 (2015).
2. Mela Osorio y cols. Impacto de la respuesta a agentes hipometilantes o quimioterapia intensiva en la sobrevida de pacientes con leucemia mieloide aguda en mayores de 65 años. HEMATOLOGÍA. Vol 23, Número Extraordinario, Suplemento 2, XXIV Congreso Argentino de Hematología.Resúmenes de trabajos científicos. O-060 (13130) (2019).
3. DiNardo, C. D. et al. Safety and preliminary efficacy of venetoclax with decitabine or azacitidine in elderly patients with previously untreated acute myeloid leukaemia: a non-randomised, open-label, phase 1b study. Lancet Oncol. 19, 216–228 (2018).
4. Wei, A. H. et al. Venetoclax Combined With Low-Dose Cytarabine for Previously Untreated Patients With Acute Myeloid Leukemia: Results From a Phase Ib/II Study. J. Clin. Oncol. 37, 1277–1284 (2019).
5. Datos provistos por laboratorio Abbvie a partir del estudio M14-358/387. Disponible por solicitud al laboratorio.
6. Tikhonova, I. A. et al. Azacitidine for Treating Acute Myeloid Leukaemia with More Than 30 % Bone Marrow Blasts: An Evidence Review Group Perspective of a National Institute for Health and Care Excellence Single Technology Appraisal. Pharmacoeconomics 35, 363–373 (2017).
7. Kantarjian, H. M. et al. Multicenter, randomized, open-label, phase III trial of decitabine versus patient choice, with physician advice, of either supportive care or low-dose cytarabine for the treatment of older patients with newly diagnosed acute myeloid leukemia. J. Clin. Oncol. 30, 2670–2677 (2012).
8. Pollyea, D. A. et al. Venetoclax in Combination with Hypomethylating Agents Induces Rapid, Deep, and Durable Responses in Patients with AML Ineligible for Intensive Therapy. Blood 132(Suppl 1), 285 (2018).
9. He, J., Xiu, L., De Porre, P., Dass, R. & Thomas, X. Decitabine reduces transfusion dependence in older patients with acute myeloid leukemia: results from a post hoc analysis of a randomized phase III study. Leuk. Lymphoma 56, 1033–1042 (2015).
10. Fenaux, P. et al. Azacitidine prolongs overall survival compared with conventional care regimens in elderly patients with low bone marrow blast count acute myeloid leukemia. J. Clin. Oncol. 28, 562–569 (2010).
