# Supplementary material for: Budget impact analysis of venetoclax for the management of acute myeloid leukemia from the perspective of the social security and the private sector in Argentina
Source: PLoS One. 2024 Jan 4;19(1):e0295798. doi: 10.1371/journal.pone.0295798 (PMC10766175; doi:10.1371/journal.pone.0295798)
Supplement: S2 File — (DOCX) [file pone.0295798.s007.docx]

*PLOS ONE*

**Budget impact analysis of venetoclax for the management of acute myeloid leukemia from the perspective of the social security and the private sector in Argentina**

Alfredo Palacios,^1,2,3^ Natalia Espinola,^1^ Juan Martin Gonzalez,^1^ Carlos Rojas-Roque,^1,3^ Maria Marta Rivas,^4^ Diego Kanevski,^5^ Pierre Morisset,^5^ Federico Augustovski,^1^ Andres Pichon-Riviere,^1^ Ariel Bardach^1^

^1^ Department of Health Technology Assessment and Health Economics, Institute for Clinical Effectiveness and Health Policy (IECS), Buenos Aires, Argentina

^2^ Department of Economics, Universidad de Buenos Aires, Buenos Aires, Argentina

^3^ Centre for Health Economics (CHE), University of York, York, UK

^4^ Hospital Universitario Austral, Buenos Aires, Argentina

^5^ AbbVie Argentina, Ing. Enrique Butty 240, C1106 CABA, Argentina

**Corresponding author**

Alfredo Palacios, MSc

Department of Health Technology Assessment and Health Economics, Institute for Clinical Effectiveness and Health Policy (IECS), Buenos Aires, Argentina

Department of Economics, Universidad de Buenos Aires, Buenos Aires, Argentina

Centre for Health Economics (CHE), University of York, York, UK

Email: alfredo.palacios@york.ac.uk

ORCID: 0000-0001-7684-0880

**Declarations**

**Conflicts of interest/Competing interests**

I have read the journal's policy and the authors of this manuscript have the following competing interests. Alfredo Palacios, Natalia Espinola, Juan Martin González, Carlos Rojas-Roque, Andrés Pichon-Riviere, Federico Augustovski and Ariel Bardach declare that they have no conflicts of interest. Diego Kanevsky and Pierre Morisset are employees of Abbvie and may own Abbvie stocks. Maria Marta Rivas has received speaker fees from Abbvie. This does not alter our adherence to PLOS ONE policies on sharing data and materials.

**Data Accessibility Statement**

All parameters used to populate the budget impact model are provided within the main manuscript and its supplementary material. The budget impact model itself will be made available upon reasonable request.

**Consent for publication**.

Not applicable.

**Acknowledgements.**

The authors wish to thank Isolda Fernandez, Mariela Gómez, Hernán Dick, Laura Fischman and Irene Rey, who participated in the modified Delphi panel to validate or adapt the model’s structure and all the parameters required to populate the budget impact model.

# **Supplementary Material S6.** General document of the declaration of conflicts of interest signed by participants in modified Delphi Panel.

**CONSENTIMIENTO INFORMADO PARA LA REUNIÓN DE CONSENSO DE EXPERTOS EN LEUCEMIA MIELOIDE AGUDA EN ARGENTINA**

**Título del estudio:** Análisis de Impacto Presupuestario (AIP) de Venetoclax en Leucemia Mieloide Aguda (LMA) de reciente diagnóstico en pacientes mayores de 65 años de edad no candidatos a inducción plena, desde la perspectiva de la Seguridad Social y el Sector Privado de salud en Argentina

**Investigadores:** Alfredo Palacios, Natalia Espinola, Juan Martín González, María Rivas, Ariel Bardach, Federico Augustovski, Andrés Pichon-Riviere

**Institución responsable:** Instituto de Efectividad Clínica y Sanitaria (IECS) – Departamento de Evaluación de Tecnologías Sanitarias y Economía de la Salud

**Financiado por:** Abbvie Argentina

1. **Introducción**

Usted ha sido invitada/o a participar en una reunión de consenso de expertos que tiene por objetivo relevar y/o validar datos epidemiológicos y del manejo de la Leucemia Mieloide Aguda de reciente diagnóstico en pacientes mayores de 65 años de edad no candidatos a inducción plena desde la perspectiva de la seguridad social y el sector privado de salud de Argentina.

Antes de comenzar con la actividad, le solicitamos por este medio que nos exprese su consentimiento para participar de la misma. Por favor, lea detenidamente la descripción de la actividad y su participación en ella que se detallan a continuación, y siéntase libre de hacer preguntas o pedir aclaración sobre cualquier punto.

1. **¿Cuál es el propósito de la actividad?**

La presente actividad tiene como objetivo relevar y/o validar datos epidemiológicos y del manejo de la Leucemia Mieloide Aguda de reciente diagnóstico en pacientes mayores de 65 años de edad no candidatos a inducción plena desde la perspectiva de la seguridad social y el sector privado de salud de Argentina.

1. **¿En qué consiste su participación?**

Usted participará, al igual que otros referentes claves, completando el cuestionario durante la reunión de consenso y siendo parte de la discusión. Las preguntas que usted y el resto de los expertos en el tema respondan se refieren a información que no ha podido ser caracterizada adecuadamente en la búsqueda y revisión de la literatura científica. Por lo tanto, apelamos a su conocimiento y experiencia en el tema para ayudar a dar respuesta a estos aspectos, necesarios para llevar adelante nuestro estudio.

1. **¿Cómo se maneja la privacidad de sus opiniones?**

La información volcada por usted en el cuestionario será tratada en forma estrictamente confidencial. En cumplimiento con la Ley 25.326 de protección de datos personales, la información que usted nos facilite no será utilizada fuera de este estudio. En todo momento usted tendrá acceso a su información personal y podrá rectificarla en caso de ser necesario. Si usted decide retirarse del estudio puede hacerlo libremente, en cualquier momento, sin sufrir ningún perjuicio y a partir de ese momento sus datos no serán utilizados en el estudio. En el caso de que se realice una presentación o publicación científica y se especifique la composición del panel de expertos en el que está participando, en ningún momento se describirán sus opiniones personales.

1. **¿Qué riesgos existen? ¿Qué beneficios obtendrá?**

No hay riesgos asociados a la participación en la actividad de consenso de expertos. No hay beneficios directos por participar en dicha actividad, aunque si usted está de acuerdo en participar, estará colaborando con nosotros en generar información relevante para la toma de decisiones en Argentina.

1. **¿Tiene algún costo su participación? ¿Recibirá algún pago por participar?**

Su participación no tiene costo alguno. El laboratorio Abbvie realizará una retribución por su participación en este consenso de acuerdo a los términos discutidos oportunamente.

***Declaración de conflictos de interés***

Por favor, detalle los potenciales conflictos de interés que podría implicar su participación en este panel de expertos:

_____________________________________________________________________________________

_____________________________________________________________________________________

_____________________________________________________________________________________

_____________________________________________________________________________________

***Declaración de conformidad***

*Por medio de la presente, comunico que:*

1. *Acepto de conformidad los términos y condiciones aquí dispuestas:* SI NO
2. *Acepto que se mencione mi nombre en el caso de que surja una publicación científica producto de este estudio (y que requiera mencionar los nombres de los expertos participantes en el evento):* SI NO

-------------------------------------------

Firma

-------------------------------------------

Aclaración

Ciudad Autónoma de Buenos Aires, 29 de noviembre de 2022

# 
